# Hypomethylation of the *MN/CA9* promoter and upregulated *MN/CA9* expression in human renal cell carcinoma

**DOI:** 10.1054/bjoc.2001.1951

**Published:** 2001-08

**Authors:** M Cho, H Uemura, S-C Kim, Y Kawada, K Yoshida, Y Hirao, N Konishi, S Saga, K Yoshikawa

**Affiliations:** Department of Urology and; Second Department of Pathology, Nara Medical University, 840 Shijo-cho, Kashihara, 634-8522, Nara; Second Department of Pathology, Aichi Medical University, Aichi, Japan

**Keywords:** *MN/CA9*, hypomethylation, renal cell carcinoma

## Abstract

*MN/CA9* is a cancer-related gene, frequently activated in human renal cell carcinomas (RCCs). To reveal the activation mechanism, we investigated the relationship between methylation status of the *MN/CA9* promoter region and gene expression using 13 human RCCs, and examined the effect of in vitro CpG methylation on the *MN/CA9* promoter activity using a human RCC cell line (SK-RC-44), expressing *MN/CA9*. *MN/CA9* expression was evaluated by RT-PCR and observed in 10 of 13 RCCs (77%). A total of 9 out of 10 *MN/CA9*-positive RCCs (90%) contained clear cell components. Methylation status of 6 CpGs in the *MN/CA9* promoter region was decided by using the bisulfite genomic sequencing protocol. Out of 13 RCCs 9 (69%) showed partial hypomethylation of the CpG at −74 bp, while the other 4 RCCs and 3 normal kidney tissue samples showed complete methylation. Hypomethylation of the CpG at −74 bp was strongly correlated with *MN/CA9* expression. Luciferase assay revealed that the *MN/CA9* promoter activity was strongly suppressed by methylation of the CpG at −74 bp. These findings suggest that hypomethylation of the CpG at −74 bp in the *MN/CA9* promoter region might play an important role in this gene activation of human RCC. © 2001 Cancer Research Campaign http://www.bjcancer.com


*British Journal of Cancer* (2001) **85**(4), 563–567
© 2001 Cancer Research Campaign
doi: 10.1054/ bjoc.2001.1951, available online at http://www.idealibrary.com on IDEAL

### Hypomethylation of the *MN/CA9* promoter and upregulated *MN/CA9* expression in human renal cell carcinoma

**M Cho[1], H Uemura[1], S-C Kim[1], Y Kawada[1], K Yoshida[1], Y Hirao[1], N Konishi[2], S Saga[3] and K Yoshikawa[3]**

[1]Department of Urology and [2]Second Department of Pathology, Nara Medical University, 840 Shijo-cho, Kashihara, Nara 634-8522; [3]Second Department of Pathology, Aichi Medical University, Aichi, Japan

**Summary** *MN/CA9* is a cancer-related gene, frequently activated in human renal cell carcinomas (RCCs). To reveal the activation mechanism, we investigated the relationship between methylation status of the *MN/CA9* promoter region and gene expression using 13 human RCCs, and examined the effect of in vitro CpG methylation on the *MN/CA9* promoter activity using a human RCC cell line (SK-RC-44), expressing *MN/CA9. MN/CA9* expression was evaluated by RT-PCR and observed in 10 of 13 RCCs (77%). A total of 9 out of 10 *MN/CA9*-positive RCCs (90%) contained clear cell components. Methylation status of 6 CpGs in the *MN/CA9* promoter region was decided by using the bisulfite genomic sequencing protocol. Out of 13 RCCs 9 (69%) showed partial hypomethylation of the CpG at –74 bp, while the other 4 RCCs and 3 normal kidney tissue samples showed complete methylation. Hypomethylation of the CpG at –74 bp was strongly correlated with *MN/CA9* expression. Luciferase assay revealed that the *MN/CA9* promoter activity was strongly suppressed by methylation of the CpG at –74 bp. These findings suggest that hypomethylation of the CpG at –74 bp in the *MN/CA9* promoter region might play an important role in this gene activation of human RCC. © 2001 Cancer Research Campaign http://www.bjcancer.com

**Keywords**: *MN/CA9*, hypomethylation, renal cell carcinoma

Human renal cell carcinoma (RCC) cells frequently express a tumour-associated antigen, MN, recognized by mouse monoclonal antibody G250 (Oosterwijk et al, 1986; Grabmaier et al, 2000). This antigen is a plasma membrane glycoprotein with an apparent molecular weight of 54/58 kDa (Pastorek et al, 1994), detectable in several types of malignancies as well as RCCs, e.g. cervical and ovarian (Liao et al, 1996), colorectal (Saarnio et al, 1998), oesophageal (Turner et al, 1997) and bladder cancers (Uemura et al, 1996). MN expression in normal tissues is restricted to the alimentary tract (Oosterwijk et al, 1986), suggesting a possible role in oncogenesis. Although MN may be useful as a target molecule for immunotherapy of RCCs (Uemura et al, 1999) and as a biomarker for kidney (Mckiernan et al, 1997), cervical (Liao et al, 1996), and colorectal cancers (Saarnio et al, 1998), its function and mechanisims of activation of the MN coding gene, *MN/CA9*, in malignancies remains poorly understood. The few investigations that have been conducted have suggested possible roles of VHL (Ivanov et al, 1998), p53 (Kaluzova et al, 2000) and DNA hypomethylation (Cho et al, 2000) in malignancies.

Inverse correlations are frequently observed between DNA methylation and gene transcription in both normal and malignant cells. CpG methylation in regulatory regions can influence transcription directly by interfering with the binding of positively or negatively acting transcription factors or indirectly by the formation of inactive chromatin (Zingg et al, 1997). DNA hypomethylation is associated with overexpression or activation of some genes linked to cancer, e.g. c-*myc* in hepatocellular carcinomas (Tsujiuchi et al, 1999), *bcl*-2 in chronic lymphocytic leukaemias (Hanada et al, 1993), *MAGE*-1 in melanomas (De Smet et al, 1996), and *MDR*1 in acute myeloid leukaemias (Nakayama et al, 1998). The expression of some tissue-specific genes, such as these for transglutaminase (Lu and Davies, 1997), L-histidine decarboxylase (Kuramasu et al, 1998) and globin (Busslinger et al, 1983) is correlated inversely with the methylation status of the promoter region. The fact that expression of *MN/CA9* is tissue specific and cancer related, suggests a possible role for DNA hypomethylation in its activation. The region immediately upstream of the transcription start site of *MN/CA9* has no TATA box and no CpG island and contains consensus sequences for transcription factors such as AP1, AP2, and p53 (Opavsky et al, 1996).

Our previous data demonstrated that *MN/CA9* activation is associated with hypomethylation of the promoter region in human RCC cell lines (Cho et al, 2000). It is interesting for us to reveal whether such a correlation would be observed in vivo and whether epigenetic change of particular CpG sites would affect *MN/CA9* transcription.

In the present study, we investigated the correlation between *MN/CA9* expression and methylation status of *MN/CA9* promoter using human RCC tissue materials and examined the effect of DNA methylation on the promoter activity of *MN/CA9*.

*Received 23 November 2000*
*Revised 14 May 2001*
*Accepted 15 may 2001*

*Correspondence to:* H Uemura

### MATERIALS AND METHODS

### RT-PCR analyses

Tissue specimens were obtained from 13 patients with RCCs who underwent radical operations in Nara Medical University Hospital.

All specimens were processed immediately after nephrectomy, and small pieces of tumour or normal kidney tissue were frozen in liquid nitrogen and stored at –80˚C. The remainder of each kidney was submitted for pathological examination. Histopathological diagnosis was conducted by one pathologist (NK). Total RNAs were isolated from frozen tissue samples by using an ISOGEN kit (NIPPON GENE, Toyama, Japan). RT-PCR was performed to estimate *MN/CA9* expression as described previously (Cho et al, 2000). PCR primers were designed to cover the 495-bp region from exon 1 to 3 of *MN/CA9* (sense: 5′-ACTGCTGCTTCTG-ATGCCTGT-3′, antisense: 5′-TCCCGCCGCTCCCAGAACT-3′). The amplification was achieved with 30 cycles, each consisting of denaturation (95˚C, 2 min), annealing (68˚C, 2 min), and extension (72˚C, 1 min). The PCR products were electrophoresed on 1% agarose gels and stained with ethidium bromide. β-actin cDNAs were amplified as the internal control.

### Bisulfite genomic sequencing protocol

Genomic DNAs were extracted from tissue samples and treated with sodium metabisulfite as described previously (Cho et al, 2000). The 5′ region (nt–142 to +267) of the bisulfite-modified *MN/CA9* gene was amplified using nested PCR. First PCR amplification was performed in 20 μl reaction mixtures containing 3 μl of bisulfite-treated genomic DNA, 10 mM Tris-HCl, pH 8.3, 50 mM KCl, 1.5 mM MgCl2, 0.5 μM each primer, 0.2 mM dNTPs, and 0.5 U Taq DNA polymerase (AmpliTaq Gold, Perkinelmer) under the following conditions: 94˚C for 1 min, 50˚C for 2 min, and 72˚C for 3 min for 5 cycles and 94˚C for 0.5 min, 50˚C for 1.5 min, and 72˚C for 1.5 min for 30 cycles (sense: 5′-TTGGTATGGGGGAGAGGGTA-3′, antisense: 5′-GGATTTATT-TAGAGAGGAGG-3′). Nested PCR amplification was similarly carried out using 0.5 μL of the first PCR reaction mixture as a template (sense: 5′-GAGAGGGTATAGGGTTAGAT-3′, antisense: 5′-AGTGAAGAGGAGGATTTATTTAG-3′). Agarose gel-purified PCR products were directly sequenced using a sequencing kit (Sequencing PRO, TOYOBO, Osaka, Japan) with primers radiolabelled with [$\gamma$-$^{32}$P] ATP. Methylation status was investigated at 6 CpG sites in the *MN/CA9* promoter region.

### Plasmid construction

The MN –1246 to + 42 fragment from a cosmid clone was amplified by PCR using adaptor primers (sense: 5′-CGGGGTAC-CTAAAGCAGAATTC, antisense: 5′-CCGCTCGAGATGCG-GCTGACT). PCR fragments were subcloned into *Kpn* I and *Xho* I sites of pGL3-Basic vector (Promega), and nested deletions were made by the exonuclease III and mung-bean nuclease method (Henikoff, 1987). Deletion mutants containing the minimal promoter region of *MN/CA9* (–158 to +42) were used in the present study (Kaluz et al, 1999).

### Cell culture, transfection and luciferase assay

Human RCC cell lines, SK-RC-10, SK-RC-12, SK-RC-14 and SK-RC-44, were grown in RPMI 1640 medium supplemented with 10% fetal calf serum. Each cell line was plated at 2 X $10^5$ cells in 35 mm culture dishes and 50–80% confluent cells were transfected with 1 μg of reporter constructs and 3 μl of FuGENE™ 6 reagent (Roche, USA). The cells were then harvested 24 h post-transfection and luciferase quantities were assayed with a luciferase assay kit (Promega) using a TR717 Microplate Luminometer (PE Biosystems, Tokyo, Japan). All transfections were carried out in 2 independent experiments and in triplicate. Luciferase quantities were displayed as percentage value of those of positive control vectors with an enhancer and promoter of SV40 (pGL3-Control Vector, Promega). Reporter vectors expressing EGFP (pIRES2-EGFP Vector, CLONTECH) were used for examination of transfection efficiency.

### In vitro DNA methylation

Bacterial methylases *Sss* I and *Hha* I were used to methylate the *MN/CA9* promoter/luciferase reporter constructs. Plasmid DNA was incubated with 3 units of *Sss* I or *Hha* I methylase per μg of DNA in 50 mM Tris-HCL, pH 7.5/10 mM EDTA/80 mM S-adenosylmethionine/5 mM 2-mercaptoethanol. All methylation reactions were carried out at 37˚C overnight. To test the samples for complete methylation, the DNA was digested with *Sss* I or *Hha* I restriction endonucleases and analyzed by agarose gel electrophoresis.

### RESULTS

### *MN/CA9* expression in RCC tissues

Table 1 summarizes histopathological findings and the results of *MN/CA9* expression of 13 RCC tissue samples. The distribution of pathological stage, grade and morphological type was as follows: pT1a in 3, pT1b in 4, pT2 in 2, pT3a in 2, pT3b in 1 and pT4 in 1, and G1 in 2, G2 in 9 and G3 in 2, and clear cell in 7, clear/granular cell in 4, granular cell in 1 and spindle cell in 1. *MN/CA9* expression was evaluated by RT-PCR and observed in 10 of 13 RCCs (77%). Correlations between *MN/CA9* expression and tumour stage and grade were not observed.

### Methylation status of the *MN/CA9* promoter region

The methylation status of 6 CpG sites in the *MN/CA9* promoter region was examined in RCCs and non-cancerous kidney tissues. A total of 9 of 13 RCCs showed partial methylation of the CpG at –74 bp, while the other 4 showed complete methylation (Table 2). Partial methylation indicates the existence of both methylated and unmethylated CpGs. The CpG at –19 bp was not determined as to methylation status because of stacking of

**Table 1** Pathological characteristics and *MN/CA9* expression in human RCC

| Sample | Stage | Grade | Morphological type | *MN/CA9* expression |
|---|---|---|---|---|
| RCC 1 | pT1b | 2 | Clear | + |
| RCC 2 | pT1b | 2 | Clear/granular | + |
| RCC 3 | pT1a | 2 | Clear/granular | + |
| RCC 4 | pT3a | 2 | Clear/granular | + |
| RCC 5 | pT4 | 3 | Spindle | – |
| RCC 6 | pT1b | 2 | Clear | + |
| RCC 7 | pT3b | 3 | Granular | – |
| RCC 8 | pT1b | 2 | Clear | + |
| RCC 9 | pT2 | 1 | Clear/granular | – |
| RCC 10 | pT1a | 2 | Clear | + |
| RCC 11 | pT3a | 2 | Clear | + |
| RCC 12 | pT2 | 1 | Clear | + |
| RCC 13 | pT1a | 2 | Clear | + |

 *© 2001 Cancer Research Campaign*

**Table 2** Methylation status of *MNCA9* promoter region in human RCC

| | | CpG site (bp) | | | | |
|---|---|---|---|---|---|---|
| **Sample** | **–74** | **–19** | **–6** | **+4** | **+13** | **+40** |
| RCC 1 | +/– | ND | + | + | + | + |
| RCC 2 | +/– | ND | + | + | + | + |
| RCC 3 | +/– | ND | + | + | + | + |
| RCC 4 | +/– | ND | +/– | +/– | +/– | + |
| RCC 5 | + | ND | + | + | + | + |
| RCC 6 | +/– | ND | + | + | + | + |
| RCC 7 | + | ND | + | + | + | + |
| RCC 8 | +/– | ND | + | + | + | + |
| RCC 9 | + | ND | + | + | + | + |
| RCC 10 | +/– | ND | +/– | +/– | +/– | + |
| RCC 11 | + | ND | + | + | + | + |
| RCC 12 | +/– | ND | + | +/– | +/– | + |
| RCC 13 | +/– | ND | + | +/– | +/– | + |
| K 1 | + | ND | + | + | + | + |
| K 2 | + | ND | + | + | + | + |
| K 3 | + | ND | + | + | + | + |

+: methylated; +/–: partially methylated (partially hypomethylated); K1, K2, K3: non-cancerous kidney tissues; ND: not determined.

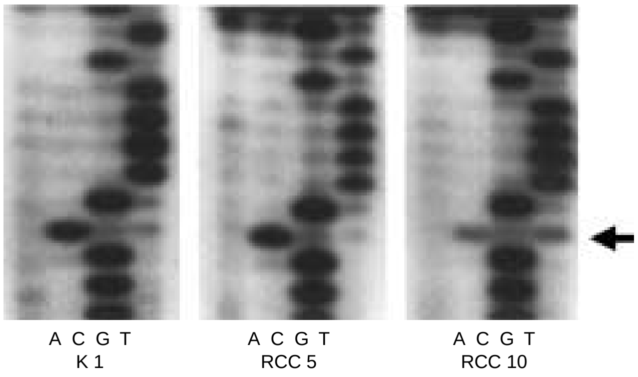

**Figure 1** Bisulfite genomic sequences. CpG at – 74 bp of *MN/CA9* was completely methylated in a non-cancerous kidney tissue (K 1) and a RCC (RCC 5), whereas partially hypomethylated in another RCC (RCC 10). Arrow shows CpG at – 74 bp sequencing gels. Other CpGs less frequently showed partial methylation in RCCs (3 out of 13). All CpGs except –19 bp were completely methylated in non-cancerous kidney tissues. Figure 1 shows bisulfite genomic sequences around the CpG at –74 bp in tissue samples.

### Correlation between *MN/CA9* expression and methylation status of the *MN/CA9* promoter region

*MN/CA9* expression was strongly correlated with partial methylation (partial hypomethylation) of the CpG at –74 bp. Out of 10 *MN/CA9*-positive RCCs (90%) showed hypomethylation, while this was the case in none of the *MN/CA9*-negative RCCs and non-cancerous kidney tissues (0%).

### Differences of *MN/CA9* promoter activities among 4 human RCC cell lines

Our previous study demonstrated that *MN/CA9* was expressed in SK-RC-10 and SK-RC-44, while not expressed in SK-RC-12 and SK-RC-14 (Cho et al, 2000). Levels of luciferase activity were 21% in SK-RC-10 cells, 13.7% in SK-RC-44 cells, 1.5% in SK-RC-12 cells and 0.6% in SK-RC-14 cells (Figure 2).

### Effects of in vitro CpG methylation on promoter activity

To examine whether CpG methylation of the *MN/CA9* promoter might influence the promoter activity, the *MN/CA9* promoter/luciferase reporter constructs were treated with *Sss* I methylase, acting all CpG sites, or with *Hha* I methylase, recognizing GCGC sequences with or without methyl donors. The *MN/CA9* promoter (–158 to + 42) contains only one GCGC sequence, corresponding to the CpG at –74 bp. Methylated and mock-methylated constructs were transfected into SK-RC-44 cells. Mock-methylated constructs with *Sss* I and *Hha* I showed luciferase activity at levels of 14.2% and 12.8%, respectively, however in neither enzyme did methylated constructs exhibit luciferase activity (Figure 3).

### DISSCUSION

The present study revealed that in human RCC tissues, hypomethylation of the CpG dinucleotide at –74 bp is strongly correlated with *MN/CA9* expression, suggesting that hypomethylation of this site may play an important role in upregulation of this marker in RCC tissues. *MN/CA9* expression did not correlate with tumour stage or grade. These findings are not consistent with our previous study (Uemura et al, 1999). The difference seems to be due to small sample size of the present study. Partial hypomethylation at this site was frequently observed in RCCs with clear cell components, while it was not in RCCs without such components and normal kidney tissues. Compared with our previous investigation using RCC cell lines (Cho et al, 2000), hypomethylation of each CpG was partial in RCC tissues. This may be explained by contamination with DNAs from non-clear cell components or surrounding non-cancerous tissues, although it cannot completely

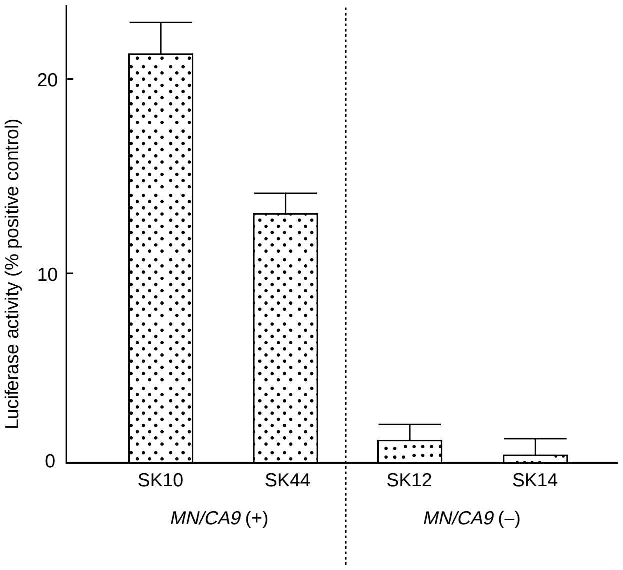

**Figure 2** Differences of *MN/CA9* promoter activities among 4 human RCC cell lines. *MN/CA9* promoter/luciferase reporter constructs were transfected into *MN/CA9* mRNA-positive cell lines (SK-RC-10 and SK-RC-44) and *MN/CA9* mRNA-negative cell lines (SK-RC-12 and SK-RC-14). *MN/CA9* promoter activities in *MN/CA9*-negative cell lines were very low compared to those in *MN/CA9*-positive cell lines

*© 2001 Cancer Research Campaign*

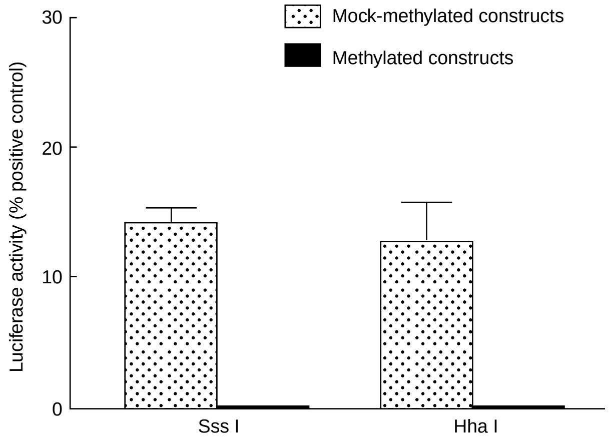

**Figure 3** Effect of CpG methylation on *MN/CA9* promoter activity. Luciferase reporter constructs containing –158/+42 bp of *MN/CA9* were treated by *Sss* I methylase acting all CpG sites or *Hha* I methylase recognizing GCGC sequence with or without S-adenosylmethionine and transfected into SK-RC-44 cells. The –158/+42 bp fragment of *MN/CA9* contains only one GCGC sequence corresponding to the CpG site at –74 bp. All CpG methylation and site-specific CpG methylation resulted in complete suppression of luciferase activity be denied that either of 2 alleles was hypomethylated in each cell of RCC tissues. Nested PCR used in the genomic sequencing protocol can make many copies from only a small amount of contaminated DNA. Another difference between RCC cell lines and RCC tissues is that hypomethylated CpGs in the *MN/CA9* promoter region were clearly sparse in RCC tissues compared to RCC cell lines. Maintenance of lines in cell culture may result in wide hypomethylation in this region.

The promoter activity of *MN/CA9* was completely lost by methylation of both whole and specific (GCGC) CpG sites of promoter/reporter constructs. The *MN/CA9* promoter (–158 to + 42) contains only one GCGC sequence, corresponding to the CpG at –74 bp. This finding, together with the strong correlation between *MN/CA9* expression and hypomethylation of the CpG at –74 bp in RCC tissues, suggests that hypomethylation of the site of the promoter may be needed to start transcription. It is well known that expression of various tissue-specific genes is associated with changes of the methylation status of their promoters (Busslinger et al, 1983; Lu and Davies, 1997; Kuramasu et al, 1998). Tumour suppressor genes are often inactivated by methylation of promoters in cancer tissues (Heman et al, 1994; Merlo et al, 1995; Yoshiura et al, 1995), while some cancer-related genes are activated by hypomethylation (Hanada et al, 1993; De Smet et al, 1996; Nakayama et al, 1998). DNA binding of several transcription factors whose recognition sequences contain a CpG is directly inhibited when the CpG is methylated (Zingg and Jones, 1997). Since the CpG at –74 bp is not located in a potential binding site for transcription factors, methylation of this site may suppress transcription by a putative methyl CpG-binding protein. Hypomethylation of oncogenes or cancer-related genes in vivo has less frequently been reported compared to hypermethylation of tumour suppressor genes. Our present data demonstrate that hypomethylation is a practically occurring and significant epigenetic change associated with gene activation in RCC tissues. It is not unclear whether hypomethylation of *MN/CA9* is caused by genome-wide hypomethylation (De Smet et al, 1996) or by gene-specific hypomethylation (Schmutte and Janes, 1998). Elucidation of the process of *MN/CA9* hypomethylation can result in discovery of other important genes contributing to the development of clear cell RCCs.

The fact that promoter activities in *MN/CA9*-negative cell lines were very low compared to those in *MN/CA9*-positive cell lines indicates the existence of specific transcription factors necessary for *MN/CA9* activation. This is consistent with our previous data. Although the expression was induced in *MN/CA9*-negative RCC cell lines (SK-RC-12 and SK-RC-14) with the demethylating agent, 5-aza-2′-deoxycitidine, the levels were much lower than in RCC cell lines with constitutive expression (Cho et al, 2000). Other authors have reported interesting findings as to *MN/CA9* expression in malignancies. *MN/CA9* was down-regulated by wt VHL in a human RCC cell line with a VHL mutation (Ivanov et al, 1998). In one recent investigation, the promoter activity of *MN/CA9* was suppressed by wt p53 in a human mammary carcinoma cell line (Kaluzova et al, 2000). Other factors such as VHL and p53 might be involved in *MN/CA9* expression in conjunction with promoter hypomethylation.

In conclusion, *MN/CA9* expression is strongly associated with hypomethylation of the specific CpG site in the *MN/CA9* promoter region in RCC tissues. However, the activation mechanism of *MN/CA9* seems to be complicated and further investigation is necessary.

### REFERENCES

Busslinger M, Hurst J and Flavell RA (1983) DNA methylation and the regulation of globin gene expression. *Cell* **34**: 197–206

Cho M, Grabmaier K, Kitahori Y, Hiasa Y, Nakagawa Y, Uemura H, Hirao Y, Ohnishi T, Yoshikawa K and Ooesterwijk E (2000) Activation of the MN/CA9 gene is associated with hypomethylation in human renal cell carcinoma cell lines. *Mol Carcinog* **27**: 184–189

De Smet C, De Backer O, Faraoni I, Lurquin C, Brasseur F and Boon T (1996) The activation of human gene MAGE-1 in tumor cells is correlated with genome-wide demethylation. *Proc Natl Acad Sci USA* **93**: 7149–7153

Grabmaier K, Vissers JL, De Weijert MC, Oosterwijk-Wakka JC, Van Bokhoven A, Brakenhoff RH, Noessner E, Mulders PA, Merkx G, Figdor CG, Adema GJ and Oosterwijk E (2000) Molecular cloning and immunogenicity of renal cell carcinoma-associated antigen G250. *Int J Cancer* **85**: 65–70

Hanada M, Delia D, Aiello A, Stadtmauer E and Reed JC (1993) bcl-2 gene hypomethylation and high-level expression in B-cell chronic lymphocytic leukemia. *Blood* **82**: 1820–1828

Henikoff S (1987) Unidirectional digestion with exonuclease III in DNA sequence analysis. *Methods Enzymol* **155**: 156–165

Herman JG, Latif F, Weng Y, Lerman MI, Zbar B, Liu S, Samid D, Duan DSR, Gnarra JR, Linehan WM and Baylin SB (1994) Silencing of the VHL tumor-suppressor gene by DNA methylation in renal carcinoma. *Proc Natl Acad Sci USA* **91**: 9700–9704

Ivanov SV, Kuzmin I, Wei MH, Pack S, Geil L, Johnson BE, Stanbridge EJ and Lerman MI (1998) Down-regulation of transmembrane carbonic anhydrases in renal cell carcinoma cell lines by wild-type von Hippel-Lindau transgenes. *Proc Natl Acad Sci USA* **95**: 12596–12601

Kaluz S, Kaluzova M, Opavsky R, Pastorekova S, Gibadulinova A, Dequiedt F, Kettmann R and Pastorek J (1999) Transcriptional regulation of the MN/CA 9 gene coding for the tumor-associated carbonic anhydrase IX. Identification and characterization of a proximal silencer element. *J Biol Chem* **274**: 32588–32595

Kaluzova M, Pastorekova S, Pastorek J and Kaluz S (2000) P53 tumour suppressor modulates transcription of the TATA-less gene coding for the tumour-associated carbonic anhydrase MN/CA IX in MaTu cells. *Biochim Biophys Acta* **1491**: 20–26

Kuramasu A, Saito H, Suzuki S, Watanabe T and Ohtsu H (1998) Mast cell-/basophil-specific transcriptional regulation of human L-histidine decarboxylase gene by CpG methylation in the promoter region. *J Biol Chem* **273**: 31607–31614

Liao SY, Aurelio ON, Jan K, Zavada J and Stanbridge EJ (1997) Identification of the MN/CA9 protein as a reliable diagnostic biomarker of clear cell carcinoma of the kidney. *Cancer Res* **57**: 2827–2831

Lu S and Davies PJA (1997) Regulation of the expression of the tissue transglutaminase gene by DNA methylation. *Proc Natl Acad Sci USA* **94**: 4692–4697

 © 2001 Cancer Research Campaign

McKiernan JM, Buttyan R, Bander NH, de la Taille A, Stifelman MD, Emanuel ER, Bagiella E, Rubin MA, Katz AE, Olsson CA and Sawczuk IS (1999) The detection of renal carcinoma cells in the peripheral blood with an enhanced reverse transcriptase-polymerase chain reaction assay for MN/CA9. *Cancer* **86**: 492–497

Merlo A, Herman JG, Mao L, Lee DJ, Gabrielson E, Burger PC, Baylin SB and Sidransky D (1995) 5′ CpG island methylation is associated with transcriptional silencing of the tumour suppressor p16/CDKN2/MTS1 in human cancers. *Nat Med* 1: 686–692

Nakayama M, Wada M, Harada T, Nagayama J, Kusaba H, Ohshima K, Kozuru M, Komatsu H, Ueda R and Kuwano M (1998) Hypomethylation status of CpG sites at the promoter region and overexpression of the human MDR1 gene in acute myeloid leukemias. *Blood* **92**: 4296–4307

Oosterwijk E, Ruiter DJ, Hoedemaeker PJ, Pauwels EK, Jonas U, Zwartendijk J and Warnaar SO (1986) Monoclonal antibody G 250 recognizes a determinant present in renal-cell carcinoma and absent from normal kidney. *Int J Cancer* **38**: 489–494

Opavsky R, Pastorekova S, Zelnik V, Gibadulinova A, Stanbridge EJ, Zavada J, Kettmann R and Pastorek J (1996) Human MN/CA9 gene, a novel member of the carbonic anhydrase family: structure and exon to protein domain relationships. *Genomics* **33**: 480–487

Pastorek J, Pastorekova S, Callebaut I, Mornon JP, Zelnik V, Opavsky R, Zat′ovicova M, Liao S, Portetelle D, Stanbridge EJ, Zavada J, Burny A and Kettmann R (1994) Cloning and characterization of MN, a human tumor-associated protein with a domain homologous to carbonic anhydrase and a putative helix-loop-helix DNA binding segment. *Oncogene* **9**: 2877–2888

Saarnio J, Parkkila S, Parkkila AK, Haukipuro K, Pastorekova S, Pastorek J, Kairaluoma MI and Karttunen TJ (1998) Immunohistochemical study of colorectal tumors for expression of a novel transmembrane carbonic anhydrase, MN/CA IX, with potential value as a marker of cell proliferation. *Am J Pathol* **153**: 279–285

Schmutte C and Jones PA (1998) Involvement of DNA methylation in human carcinogenesis. *Biol Chem* **379**: 377–388

Tsujiuchi T, Tsutsumi M, Sasaki Y, Takahama M and Konishi Y (1999) Hypomethylation of CpG sites and c-myc gene overexpression in hepatocellular carcinomas, but not hyperplastic nodules, induced by a choline-deficient L-amino acid-defined diet in rats. *Jpn J Cancer Res* **90**: 909–913

Turner JR, Odze RD, Crum FCP and Resnick MB (1997) MN antigen expression in normal, preneoplastic, and neoplastic esophagus: a clinicopathological study of a new cancer-associated biomarker. *Human Pathol* **28**: 740–744

Uemura H, Kitagawa H, Hirao Y, Okajima E, Debruyne FMJ and Oosterwijk E (1997) Expression of tumor-associated antigen MN/G250 in urologic carcinoma: potential therapeutic target. *J Urol Suppl* **157**: 377

Uemura H, Nakagawa Y, Yoshida K, Saga S, Yoshikawa K, Hirao Y and Oosterwijk E (1999) MN/CA IX/G250 as a potential target for immunotherapy of renal cell carcinomas. *Br J Cancer* **81**: 741–746

Yoshiura K, Kanai Y, Ochiai A, Shimoyama Y, Sugimura T and Hirohashi S (1995) Silencing of the E-cadherin invasion-suppressor gene by CpG methylation in human carcinomas. *Proc Natl Acad Sci USA* **92**: 7416–7419

Zingg JM and Jones PA (1997) Genetic and epigenetic aspects of DNA methylation on genome expression, evolution, mutation and carcinogenesis. *Carcinogenesis* **18**: 869–882

*© 2001 Cancer Research Campaign*
